# Impact of immunosuppressive therapy on pulmonary perfusion in kidney transplant recipients after COVID-19 illness

**DOI:** 10.3389/fmed.2025.1562407

**Published:** 2025-06-11

**Authors:** Barbara Infante, Dario Troise, Matteo Gravina, Bruno Minopoli, Marcella Gambacorta, Carmen Montanile, Luca Macarini, Silvia Mercuri, Annalisa Cappiello, Maddalena Panico, Elena Ranieri, Giuseppe Stefano Netti, Francesca Fortunato, Carlo Alfieri, Giuseppe Castellano, Giovanni Stallone

**Affiliations:** ^1^Nephrology, Dialysis and Transplantation Unit, Department of Medical and Surgical Sciences, Advanced Research Center on Kidney Aging (A.R.cK.A), University of Foggia, Foggia, Italy; ^2^Renal Medicine and Baxter Novum, Department of Clinical Science, Intervention and Technology, Karolinska Institutet, Stockholm, Sweden; ^3^Radiology Unit, Department of Medical and Surgical Sciences, University of Foggia, Foggia, Italy; ^4^Clinical Pathology Unit and Center for Molecular Medicine, Department of Medical and Surgical Sciences, Advanced Research Center on Kidney Aging, University of Foggia, Foggia, Italy; ^5^Hygiene Unit, Department of Medical and Surgical Sciences, University of Foggia, Foggia, Italy; ^6^Department of Clinical Sciences and Community Health, University of Milan Fondazione IRCCS Cà' Granda Ospedale Maggiore Policlinico, Milan, Italy

**Keywords:** immunosuppression, kidney transplantation, COVID-19, pulmonary perfusion, mTOR inhibitors

## Abstract

**Introduction:**

Patients who have received kidney transplants (KTR) are considered to be more susceptible to the severity of COVID-19-related illness. The transplanted patient’s respiratory outcome worsened because of the ventilation-perfusion mismatch that occurs during the infection, which has been linked to endothelial damage. In this context, a reduction in immunosuppressive therapy is advisable to improve patient outcomes. However, the prognosis and suggested treatment for these types of patients are still debated.

**Methods:**

We retrospectively analyzed 48 KTRs with stable graft function on calcineurin inhibitor therapy who underwent transient modification of the maintenance immunosuppressive regimen with withdrawal of mycophenolic acid/mycophenolate mofetil or mTOR inhibitor (mTORi) during COVID-19 infection and their reintroduction after healing. Pulmonary functional tests (EGA and spirometry) and DECT (Dual-energy CT) scans were performed 1 month following the negative nasopharyngeal swab (T0) and then after 6 months (T6).

**Results:**

The presence an mTOR inhibitor in immunosuppressive therapy was associated with a significant increase in lung perfusion for the entire lung parenchyma of the mTORi-treated group, both in each lung segment considered separately and all of them together.

**Conclusion:**

Our findings are consistent with the observation that the use of mTORi could play a potentially beneficial role in improving pulmonary perfusion.

## 1 Introduction

Coronavirus-2019 disease (COVID-19) has caused over 767 million confirmed cases and more than 6.9 million deaths worldwide (1). The pathophysiology of SARS-CoV-2 infection is characterized by a systemic proinflammatory state mediated by the release of several cytokines, a condition known as “cytokine storm,” which cause damage to the entire body. The virus enters the human system through the airways delivered by droplets and aerosols or by direct contact, reaching the alveolar tissue where it binds to angiotensin-converting enzyme 2 receptors via spike protein, allowing its access into the cells and resulting in cellular damage (2).

COVID-19 virus disease is characterized by coagulation disorders driven by the cytokine storm, which leads to microvascular thrombosis in the lungs and therefore, ventilation-perfusion mismatch, which in association with inflammatory and infectious alterations worsen the patient’s respiratory outcome (3). In this context, it is important to pay attention to the effect of COVID-19 infection on the pulmonary circulation because there is evidence that endothelial injury, capillary thrombi, and new vessel formation occur during acute COVID-19 infection (4).

Although reverse transcription polymerase chain reaction (RT-PCR) is required for the diagnosis of SARS-CoV-2 infection in all affected patients, instrumental investigations, including lung imaging, such as chest X-ray and computed tomography (CT), have become a valid diagnostic tool for the management of the disease. On the other hand, Chest X-ray frequently fails to identify infection-related radiological patterns and vascular involvement, especially ground-glass opacities, which represent the most common imaging pattern of the disease, while they are usually visible on CT scans, suggesting that CT is the most sensitive instrumental investigation (5).

Due to its capacity to show variations in organ blood flow, dual-energy computed tomography (DECT) has been proposed as a useful tool for the detection of pulmonary perfusion abnormalities and pulmonary embolism in COVID-19 pneumonia (6).

In depth, DECT can acquire images with improved vascular contrast, allowing the identification of regional lung perfusion defects using X-rays with two different energy spectra at low and high kV to generate an iodine map and reducing the required volume of iodinated contrast material, without decreasing the image quality (7).

Kidney transplant recipients (KTRs) affected by SARS-CoV-2 infection are considered to be at a higher risk patient for severe disease with increased mortality and morbidity (8) because they are subjected to long-term immunosuppression in order to prevent organ rejection. Therefore, the prognosis and suggested treatment for these patients are still debated (9). Studies have shown that a reduction in immunosuppressive therapy can improve patient outcomes (10) and withdrawal of anti-proliferative drugs, such as mycophenolate mofetil (MMF), mycophenolic acid (MPA), azathioprine and mTOR inhibitors (mTORi), such as sirolimus and everolimus, is recommended during COVID-19 infection (11). Lack of evidence exists regarding how and when to restore immunosuppressive therapy and which drugs to use to minimize the effects of prior COVID-19 infection in this patient’s setting. Therefore, in this study, we investigated whether immunosuppressive therapy in KTRs with previous COVID-19 infection, might have an influence on respiratory outcomes, focusing on pulmonary function and perfusion.

## 2 Materials and methods

### 2.1 Study participants and design

We conducted a retrospective observational single-center study. The study protocol was approved by the local Ethical Committee “Interprovincial Ethics Committee Area 1 – University Hospital of Foggia, ASL FG, ASL BAT” (N. 177–2023). All patients had received two doses of the COVID-19 vaccine prior to the start of the study. All the enrolled patients at COVID-19 infection diagnosis underwent transient modification of the maintenance immunosuppressive regimen until SARS-CoV-2 RT-PCR at nasal swab was negative. In detail, inclusion criteria were: age 18 years or older; history of renal transplantation performed more than 12 months prior; and maintenance immunosuppressive therapy consisting of a calcineurin inhibitor (CNI) combined with either mycophenolate mofetil/mycophenolic acid (MMF/MPA) or an mTOR inhibitor (mTORi). Furthermore, no patient was affected by underlying chronic pulmonary diseases. The patients were on therapy with calcineurin inhibitors (CNI, Tacrolimus) and MMF/MPA, or with CNI and mTORi (Everolimus) according to the immunosuppressive policy of our Transplant Center. No changes in immunosuppressive therapy were done during the last 2 years. Then, during the COVID-19 infection, all patients underwent a lowered immunosuppressive regimen with withdraw of MMF/MPA or mTORi. Upon COVID-19 recovery, the former maintenance immunosuppressive therapy was restored for both groups and all the enrolled patients were strictly followed at Nephrology, Dialysis, and Transplant Unit outpatient service.

**Figure 1:**
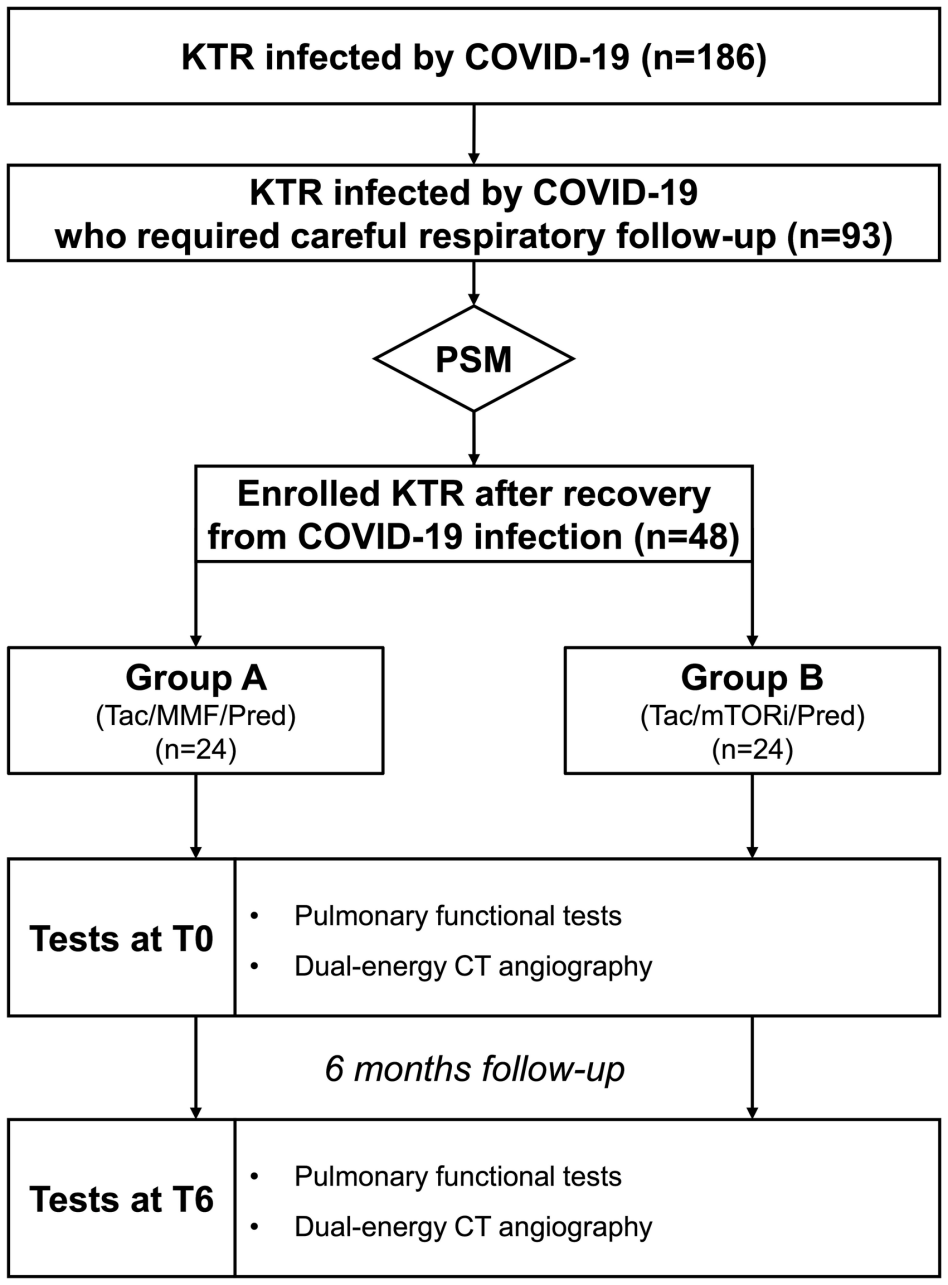
Algorhythm of the Study. KTR, kidney transplant recipients; MMF, mycophenolate mofetil; mTOR, mTOR inhibitors; Pred, prednisolon; PSM, propensity score matching; Tac, tacrolimus.

Thirty days after the negative test, all the enrolled patients underwent clinical and laboratory examinations. Both accurate pulmonary functional tests and dual-energy CT-scan were performed 1 month after the negative test (T0) and after a further 6 months (T6), as shown in Figure 1.

### 2.2 Laboratory and respiratory tests

RT-PCR was used to confirm the diagnosis of SARS-CoV-2 infection in each patient. After recovery from COVID-19 infection, all patients underwent spirometry and arterial blood gas analysis at study enrollment (T0) and after 6 months (T6). According to the recommendations of the American Thoracic Society/European Respiratory Society recommendations (12), spirometry in a sitting position was used to assess respiratory capacity. Spirometric measurements included the FEV1/FVC ratio, which is the ratio between the forced expiratory volume in 1 s (FEV1) and the forced vital capacity (FVC), which is the maximal amount of air that can be forcibly exhaled from the lungs after full inspiration. Normal breaths were taken with the mouthpiece in place, followed by a deep full breath and a quick full expiration. Three valid maneuvers were performed, and the best was chosen among them.

Blood gas analysis was performed by sampling blood from the radial or brachial artery to measure oxygen saturation (SO2), partial pressure of oxygen (PO2) and partial pressure of carbon dioxide (PCO2) using bench-top analyzers located in the Nephrology Department.

### 2.3 Thoracic imaging and analysis

All CT scans were acquired on a dedicated dual-source machine (GE Revolution EVO 256) before and after administration of the iodinated contrast media (Iodixanol 320 mg/mL, 70 mL with a flow of 3 mL/s).

The iodine maps were examined with AW SERVER 3.2 Ext.4.0 ASIST TOOL software, to quantify iodine particle passage through the examined lung parenchyma in order to study perfusion in the targeted area of interest. Dual-energy quantification allows not only to characterize lesions by measuring Hounsfield Unit (HU), but also with the amount of iodine in tissue in mg/dL.

The study was carried out using iodine maps in order to assign a numerical value to the perfusion of the lung parenchyma, and not by just densitometric and visual evaluations. A colorimetric window has also been standardized for a visual assessment of the perfusion of tissues. A colorimetric window has also been standardized for a visual assessment of tissue perfusion.

Ten regions of interest (ROIs), two per lung lobe (anterior portions and posterior segments), were used for perfusion analysis. ROIs measuring 150 mm^2^ (+/−10 mm^2^), were positioned in the upper, middle, and lower lung areas of the axial scans, avoiding the superimposition of extra-pulmonary structures (bone, pleura, diaphragm) trying to include only a single broncho-alveolar segment. Measurements were taken at the same level in both CT patient’s exams, and ROIs were placed in the same points and DECT scan perfusion (mg/dl) were recorded. The radiologists were unaware of which patients were undergoing therapy at the time of the second CT scan.

### 2.4 Statistical analysis

Statistical analysis was performed using Statistical Package for Social Sciences (SPSS) 25.0 software (SPSS Inc., Evanston, IL, United States). To enroll the patients receiving different immunosuppressive regimes who met the inclusion criteria, a propensity score matching analysis was conducted in R using the MatchIt package with nearest neighbor 1:1 matching to compare the MMF-based cohort with mTORi-based cohort. Specifically, nearest-neighbor 1:1 matching without replacement was performed using a caliper width of 0.2 times the standard deviation of the logit of the propensity score. The following clinically relevant variables were included in the propensity score model: gender, age, time from transplantation, eGFR, and presence of diabetes mellitus. A Love Plot diagram displaying standardized mean differences (SMDs) of covariates before and after propensity score matching was generated using R. An SMD < 0.1 was considered indicative of adequate balance between groups. Shapiro–Wilk test was employed to assess the normality of variable distributions. For paired comparisons between Group A and Group B at T0 as well as between T0 and T6 within each group, the Wilcoxon signed-rank test was used for non-normally distributed variables. For normally distributed variables, paired *t*-tests were applied. Categorical variables were compared using the chi-square (*X*^2^) test, as appropriate. When multiple comparisons across lung segments were performed, the Benjamini–Hochberg procedure was applied to control for false discovery rate (FDR). To account for within-subject correlation, perfusion values were averaged across all segments for each patient. Since perfusion values were averaged per subject, multiple comparisons were avoided and no correction was applied. Data are presented as mean ± standard deviation (SD) for normally distributed variables (age, time from transplantation, eGFR), median and interquartile range (IQR) for non-normally distributed variables (DECTPA mg/dl), or percentage frequencies (female gender, diabetes mellitus), unless otherwise specified. A *p*-value <0.05 was considered statistically significant.

## 3 Results

### 3.1 Demographic data

In the present study, we conducted a retrospective observational single-center study encompassing 300 consecutive KTRs actively followed at the Nephrology, Dialysis and Transplant Unit of University Foggia (Italy), between March 2021 and December (10). Among them, 184 (61.33%) were infected by COVID-19 and 93 (50.54%) – hereafter referred to as the “recruited cohort”- developed COVID-19-related respiratory syndrome and required careful respiratory follow-up. Within the recruited cohort, 48 patients were selected for the final analysis after propensity score matching and divided into two groups according to the immunosuppressive regimen (MMF-based, *n* = 24 versus mTORi-based, *n* = 24). After COVID-19 infection diagnosis, all the patients underwent transient lowering of the maintenance immunosuppressive regimen. Since COVID-19 recovery (mean time from diagnosis to virus clearance 22.1 ± 6.3 days), the former maintenance immunosuppressive therapy was restored for both groups. In detail, In Group A (Tac/MMF/Pred), Tacrolimus though levels reached 6.2 ± 2.3 ng/mL while in Group B (Tac/mTor-i/Pred) Tacrolimus through levels reached 4.8 ± 1.7 ng/mL, before and during the 6-months follow-up. At the withdrawal and at the reintroduction, MMF dosage were 500 mg bid in Group A, while Everolimus though levels were maintained around 2.9 ± 1.2 ng/mL in Group B.

As shown in Table 1, the study population included 36 males and 12 females with a mean age of 53.5 years and a mean time from transplantation of 85.3 months; there were 5 (10.4%) affected by diabetes mellitus. We did not observe statistical differences between the two groups of patients at baseline regarding age, time from transplantation, eGFR and for the presence of diabetes mellitus. They differed only in the maintenance immunosuppressive therapy (Group A: Tac/MMF-MYF/Pred; Group B: Tac/mTORi/Pred), as expected. No patients experienced kidney rejection in the 6 months prior to the COVID-19 infection or throughout the study period. There were no statistical differences between the two groups regarding the eGFR measured after the 6-months follow-up (Group A: 47.5 ± 9.6 vs. Group B: 50.1 ± 11.1, *p* = 0.220).

**Table 1:** Baseline clinical and laboratory characteristics of kidney transplant recipients enrolled in the study.

	Total	Group A (Tac/MMF/Pred)	Group B (Tac/mTORi/Pred)	*P*
Number (n)	48	24	24	
Gender (% female)	12 (25%)	6 (25%)	6 (25%)	0.630
Age (years)	53.5 ± 12.0	56.2 ± 9.3	50.7 ± 13.9	0.115
Time from transplantation (months)	85.3 ± 42.1	95.4 ± 39.9	75.2 ± 42.8	0.099
eGFR (ml/min)	50.2 ± 22.1	52.1 ± 21.7	48.3 ± 17.4	0.391
Diabete Mellitus (%)	5 (10.4%)	2 (8.3%)	3 (12.5%)	0.636
Maintenance therapy				
Tac^a^/MMF^b^/Pred	24	24	0	
Tac^a^/mTORi^c^/Pred	24	0	24	

Values are expressed as mean ± SD, counts (n), or percentages (%).

eGFR, estimated glomerular filtration rate; MMF, mycophenolate mofetil; mTOR, mTOR inhibitors; Pred, prednisolon; Tac, tacrolimus.

^a^The trough level of tacrolimus during follow-up was 5.0–7.0 ng/mL.

^b^Mycophenolate mofetil (MMF) was administered at a standard dose of 500 mg twice daily.

^c^The trough level of m-TOR-I (Everolimus) during follow-up was 3.0–5.0 ng/mL.

### 3.2 Respiratory evaluation

When we analyzed pulmonary functional tests, we did not observe any difference regarding pO2, pCO2, sO2, FEV1/FVC between kidney transplant recipients treated with MMF/MPA-based therapy and those treated with mTORi-based therapy before (T0) and after (T6) 6 months follow-up from the restoration of immunosuppressive therapy with MMF/MPA (Group A) or mTORi (Group B). This result underlines that both groups did not statistically differ in terms of the severity of the COVID-19 related lung condition and of requirement for support ventilation (Table 2).

**Table 2:** Pulmonary functional tests before (T0) and after (T6) 6 months follow up of immunosuppressive therapy with MMF/MYF or mTOR-i in kidney transplant recipients enrolled in the study.

	Group A (Tac/MMF/Pred)			Group B (Tac/mTORi/Pred)			Group A vs. B at T0
	T0	T6	*P*	T0	T6	*P*	*P*
pO2 (mmHg)	105 ± 12	108 ± 19	0.087	104 ± 15	106 ± 17	0.124	0.378
pCO2 (mmHg)	40 ± 3	38 ± 4	0.247	39 ± 2	37 ± 3	0.311	0.094
sO2 (%)	99.1 ± 0.7	99.0 ± 09	0.991	99.3 ± 0.9	99.4 ± 0.6	0.913	0.989
FEV1/FVC (%)	94.0 ± 15.1	95.9 ± 14.3	0.893	93.0 ± 13.8	95.6 ± 18.8	0.574	0.651

Values are expressed as mean ± SD.

FEV1/FVC, ratio between the forced expiratory volume in 1 s (FEV1) and the forced vital capacity (FVC).

### 3.3 Lung parenchymal findings

Next, we analyzed post COVID-19 residual findings (Table 3) at CT-scan follow-up. Lung parenchymal abnormalities consisted of a variable association of ground glass opacities, parenchymal distortion and interstitial linear, reticular opacities. No statistical difference was observed between both groups at follow-up chest CT scans at T0 and T6, and the lung parenchymal findings did not significantly improve compared to baseline evaluation.

**Table 3:** Post COVID-19 residual findings at CT-scan before (T0) and after (T6) 6 months follow up of immunosuppressive therapy with MMF/MYF or mTOR-i in kidney transplant recipients enrolled in the study.

	Group A (Tac/MMF/Pred)			Group B (Tac/mTORi/Pred)			Group A vs. B at T0
	T0	T6	*P*	T0	T6	*P*	*P*
Ground glass opacities	4 (16.7%)	3 (12.5%)	0.682	2 (8.3%)	1 (4.2%)	0.551	0.296
Parenchymal distortion	9 (37.5%)	6 (25.0%)	0.350	9 (37.5%)	11 (45.8%)	0.558	0.131
Interstitial linear/reticular opacities	8 (33.3%)	6 (25.0%)	0.525	3 (12.5%)	2 (8.3%)	0.636	0.121

Values are expressed as counts (n) or percentages (%).

GFR, glomerular filtration rate; MMF, mycophenolate mofetil; mTOR, mTOR inhibitors; Pred, prednisolon.

### 3.4 Lung perfusion findings

**Figure 2:**
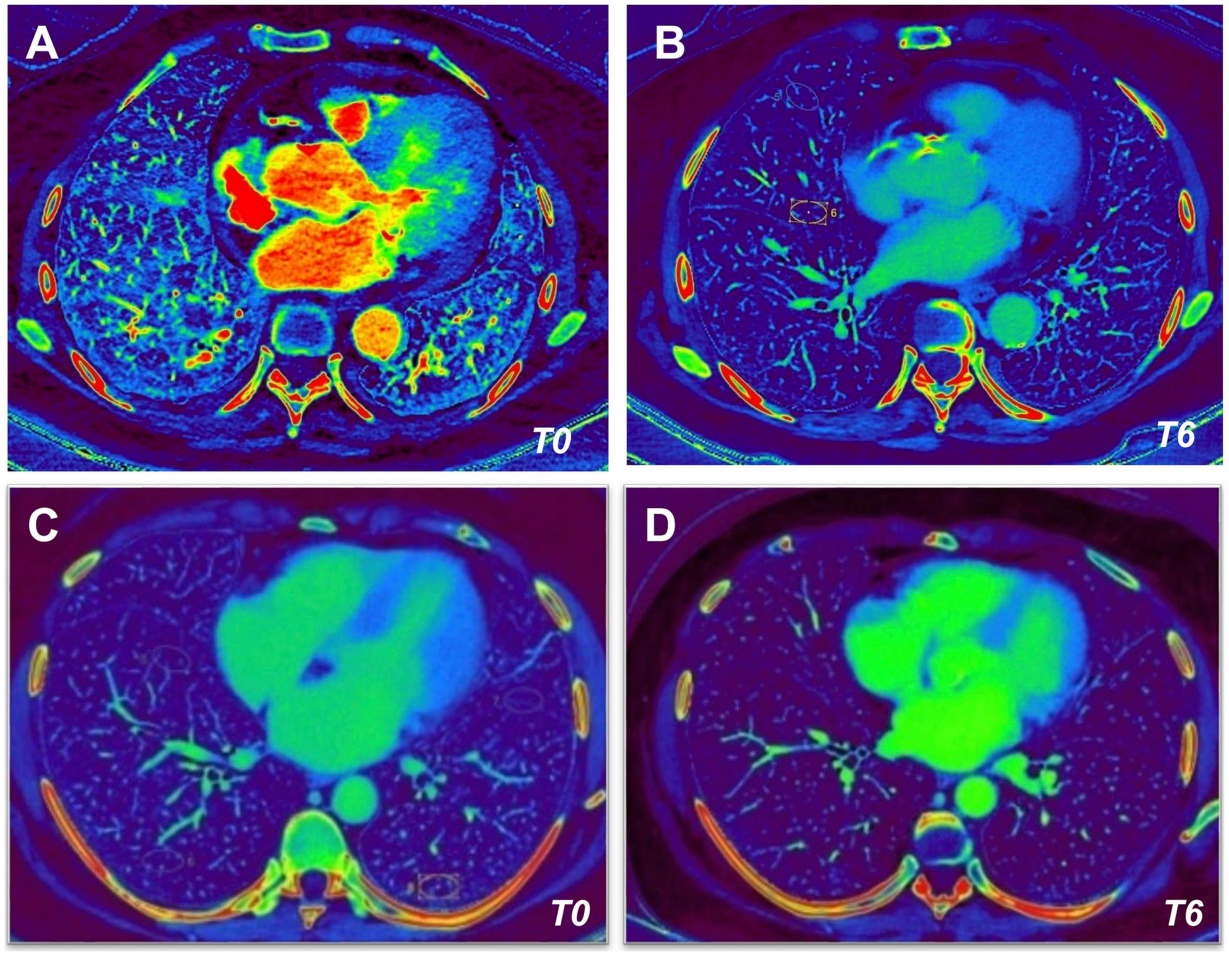
Dual-energy CT (DECT) angiography in kidney transplant recipients. Dual-Energy CT angiography obtained before (T0, left images) and after (T6, right images) 6 months follow up of immunosuppressive therapy with Tac/MMF/Pred **(A,B)** or Tac/mTORi/Pred **(C,D)** in kidney transplant recipients.

Finally, the pulmonary perfusion analysis was performed before (T0) and after (T6) 6 months follow up in all patients in both groups (Figure 2).

**Figure 3:**
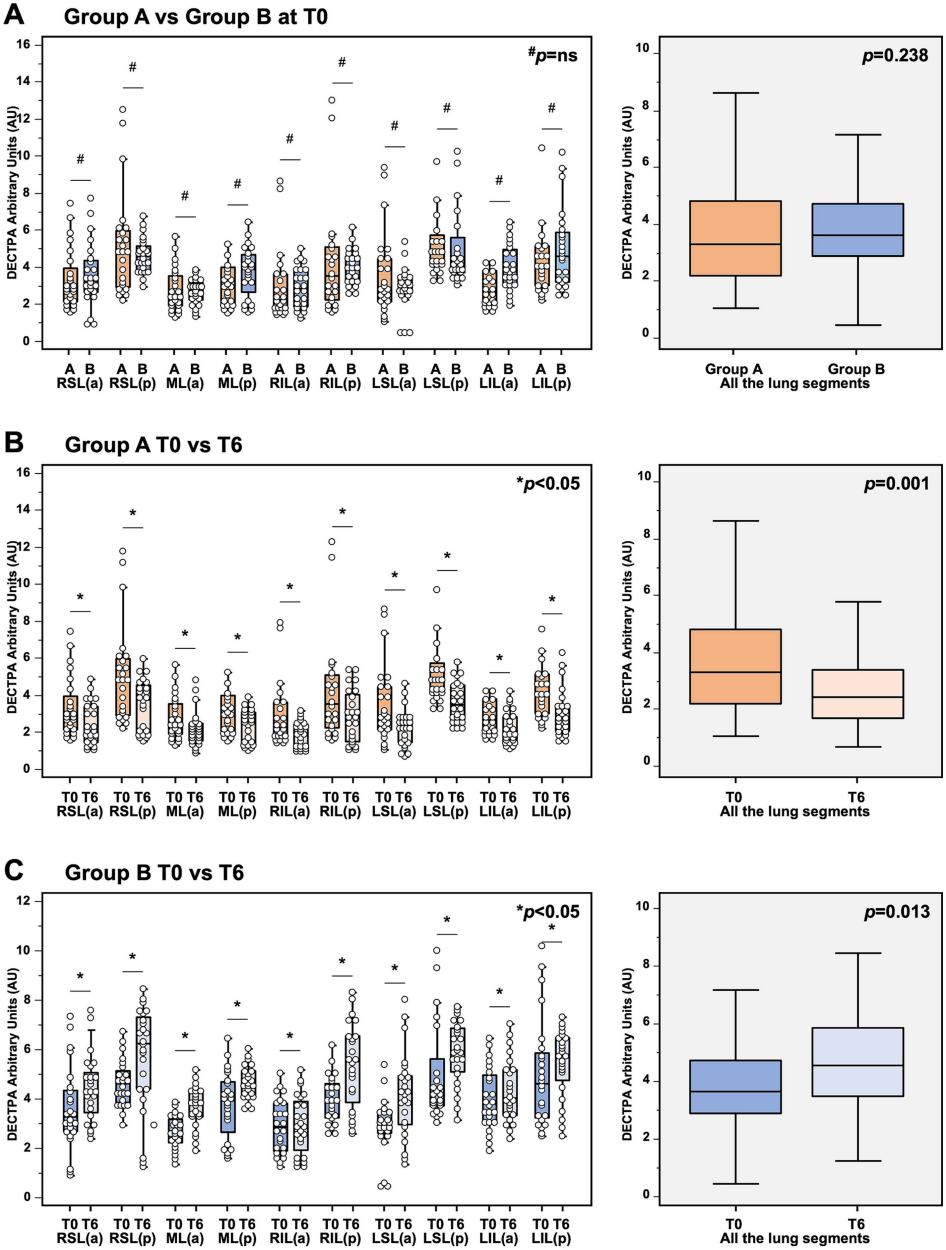
Analysis of dual-energy CT (DECT) angiography in **(A)** analysis of DECT angiography in kidney transplant recipients 1 month after COVID-19 recovery, showing no significant differences of lung perfusion between Group A (MMF-based) and Group B (mTORi-based) at baseline. In detail, no significant differences in the lung perfusion were observed if the analysis were performed in the entire lung parenchyma (3.44 [IQR 2.46–4.96] vs. 3.46 [IQR 2.83–4.41] DECTPA [mg/dl] for MMF/MYF-treated group and mTORi-based group respectively, *p* = 0.238; boxplots in the right box), as well as at each lung segment (*p* = *not significant* for each segment for MMF/MYF-treated group vs. mTORi-based group; left box). **(B)** Analysis of DECT angiography in kidney transplant recipients receiving MMF/MYF-treatment before (T0) and after 6-months follow-up (T6). In detail, a significant reduction of lung perfusion was observed if the analysis were performed in the entire lung parenchyma (3.44 [IQR 2.46–4.96] vs. 2.59 [IQR 1.78–3.52] DECTPA mg/dl for MMF/MYF-treated group at T0 and T6 respectively, *p* = 0.001; boxplots in the right box), as well as at each lung segment (*p* < 0.05 for each segment for MMF/MYF-treated group at T0 vs. T6 respectively; left box). **(C)** Analysis of DECT angiography in kidney transplant recipients receiving mTORi-treatment before (T0) and after 6-months follow-up (T6). In detail, a significant increase of lung perfusion was observed if the analysis were performed in the entire lung parenchyma (3.46 [IQR 2.83–4.41] vs. 4.43 [IQR 3.53–5.79] DECTPA mg/dl for mTORi-treated group at T0 and T6 respectively, *p* = 0.013; boxplots in the right box), as well as at each lung segment (*p* < 0.05 for each segment for mTORi-treated group at T0 vs. T6 respectively; left box). ^#^*p* = not significant; *p < 0.05; ^§^each boxplot in the right panels represents the median ± IQR of the DECT values, assessed for each patient for each lung segment examined, cumulatively, while whiskers represent 5th and 95th percentiles. RSL(a), right superior lobe – anterior area; RSL(p), right superior lobe – posterior area; ML(a), middle lobe – anterior area; ML(p), middle lobe – posterior area; RIL(a), right inferior lobe – anterior area; RIL(p), right inferior lobe – posterior area; LSL(a), left superior lobe – anterior area; LSL(p), left superior lobe – posterior area; LIL(a), left inferior lobe – anterior area; LIL(p), left inferior lobe – posterior area.

As shown in Figure 3A, we observed no significant differences between Group A and Group B at the analysis of the lung perfusion in the entire lung parenchyma at baseline (3.44 [IQR 2.46–4.96] vs. 3.46 [IQR 2.83–4.41] DECTPA [mg/dl] for MMF/MYF-treated group and mTORi-based group respectively, *p* = 0.238). No significant differences of lung perfusion signal between both groups were observed if the analysis was also performed at each lung segment.

However, as shown in Figures 3B,C, the comparative analysis of DECT scan within each group at baseline and after 6 months follow-up showed a significant reduction of perfusion in patients treated with MMF/MPA (3.44 [IQR 2.46–4.96] vs. 2.59 [IQR 1.78–3.52] DECTPA mg/dl for the entire lung parenchyma of MMF/MPA-treated group at T0 and T6 respectively, *p* = 0.001;) and a significant increase of lung perfusion in patients treated with mTORi (3.46 [IQR 2.83–4.41] vs. 4.43 [IQR 3.53–5.79] DECTPA mg/dl for the entire lung parenchyma of mTORi-treated group at T0 and T6 respectively, *p* = 0.013). This contrasting trend of the lung perfusion after 6 months of therapy with MMF/MPA as compared to mTORi was confirmed also if the analysis was performed both considering each lung segment and considering all of them together. These results highlight the influence that an mTORi-based immunosuppressive protocol can have on lung microcirculation in a cohort who do not exhibit unequivocal signs of existing fibrosis.

## 4 Discussion

In this study we observed that mTORi used as maintenance immunosuppressive therapy in kidney transplant patients who experienced a COVID-19 infection, could contribute to increase the pulmonary perfusion during recovery after the infection. Our results acquired considerable significance because they have been obtained in a fragile population, such as transplanted recipients. Management of the COVID-19 sequelae in such high-risk population is challenging because of their compromised immune status. To prevent kidney allograft rejection, transplant recipients often need a lifetime combination of three immunosuppressive medications, each with different mechanisms of action. The evidence that is currently available regarding their effects on coronaviruses replication is limited (13).

*In vitro* experiments, MPA has been shown to inhibit the proteolytic activity of Middle East Respiratory Syndrome Coronavirus and Severe Acute Respiratory Syndrome Coronavirus but it has also cytostatic effects on B and T lymphocytes, which could determine the worst outcome in case of coronavirus infections (14, 15). Moreover, the inhibition of the PI3K-AKT–MTOR pathway by mTORi, which is required for intracellular viral replication, could faster virus clearance (16) although their use would seem to be associated with a higher rejection rate compared to the use of MPA (13).

It is clear that immunosuppression is a risk factor for severe life-threatening infection in COVID-19 transplant recipient, leading to an increased morbidity and mortality. As a consequence, reduction of immunosuppression is advisable in clinical practice (17) and temporary suspension of antiproliferative drugs is recommended (11).

These findings support our clinical approach to kidney transplanted patients with COVID-19 infection, which consists of suspension of MMF/MPA/mTORi at the moment of positive nasopharyngeal swab for COVID-19 and their reintroduction after they have healed.

Spirometry is a widely used test to evaluate lung function for both diagnosis and chronic lung disease monitoring (18). Some studies have shown persistent impairment of pulmonary function tests after recovery from COVID-19 (19), whereas others, such as the study carried out by Liang et al. (20), showed that after healing and 60 days after the onset of symptoms, only a small percentage of patients showed pathological changes in spirometry, and most of these were among those who experienced severe COVID-19 pneumonia.

We observed that none of the patients considered developed pathological changes at the spirometry test performed for follow-up in our recovered COVID-19 KTRs. The reason for these results could be that the patients considered in this observational study developed moderated respiratory symptoms (sore throat, persistent cough and shortness of breath accompanied by mucus production) which may not have caused substantial alteration in lung mechanics or gas exchange. Moreover, strict monitoring likely helped to avoid the deterioration of pulmonary function. It is also important to note that none of the patients had an underlying pulmonary disease that could have significantly impacted their respiratory tests. We believe that the laboratory and respiratory tests and the pulmonary function tests assess different aspects of patient’s respiratory health, and the absence of correlation between them highlights the complexity of respiratory responses in the context of COVID-19 infection.

Several pieces of evidence demonstrate that severe hypoxemia in COVID-19-affected patients results from two main mechanisms: alveolar damage and perfusion reduction. This leads to a ventilation-perfusion mismatch, a condition in which a pulmonary area is ventilated but not perfused or vice versa (21). As the viral load increases, injured alveolar epithelial cells allow the coronavirus to infect alveolar capillary endothelial cells at the blood-air barrier. In an attempt to eliminate the virus, the enhanced defense system will inevitably cause tissue damage, leading to the destruction of weak parts of the alveolar membrane and therefore pulmonary edema. Moreover, the coagulation cascade system initiates because of endothelial cell injury with the formation of pulmonary microthrombi, which is expressed as pulmonary perfusion reduction (22).

Moreover, angiotensin-converting enzyme 2 (ACE2) is downregulated when alveolar cells are infected by SARS-CoV-2, resulting in overactivation of the ACE 1/angiotensin II/angiotensin type 1 receptor pathway, which causes the release of endothelin-1, a potent vasoconstrictor and, because of downregulation of ACE2, the inhibition of nitric oxide release, with severe pulmonary vasoconstriction as a consequence (23).

Although chest X-rays and lung ultrasonography are valuable diagnostic imaging tools, they cannot evaluate the pulmonary perfusion. Advanced imaging techniques, such as DECT, are useful for observing perfusion abnormalities in COVID-19 (6). Because of the reduction of the iodinated contrast material volume required it may reduce the possibility of contrast-induced nephropathy. Therefore, we have chosen this as the radiological examination of choice for all the kidney transplanted patients who have been infected by COVID-19 and are in active follow-up at our transplantation center.

SARS-CoV-2 is also involved in the development of pulmonary fibrosis that is a consequence of the tissue repair responses that arise after repeated tissue damage under conditions of chronic inflammation. The coronavirus has a role in fibrosis both for continuous tissue injuries mediated by persistent viral–ACE2 receptor interaction leading to abnormal and irregular healing, and for the release of profibrotic factors, like transforming growth factor *β* (TGF-β), tumor necrosis factor *α* (TNF-α), insulin-like growth factor-1 (IGF-1), platelet-derived growth factor (PDGF), connective tissue growth factor (CTGF), and extracellular matrix deposition which collectively activate lung fibroblasts and start pulmonary fibrosis. At the molecular level, the progression of fibrosis is supported by a trans-differentiation of epithelial cells into mesenchymal cells, a process known as epithelial-to-mesenchymal transition (EMT) (24). This is an important viewpoint because both antimetabolite drugs and mTORi have a toxicity profile (25). Moreover, mTORi also have a pulmonary dose-dependent toxicity probably correlated with the so-called EMT leading to tissue fibrosis, lungs included (26).

However, mTORi showed an antifibrotic effect whether a low dosage is administered. The reported antifibrotic effects of mTORi can be partially attributed to TGFβ inhibition. These findings are consistent with the effects of mTOR inhibition in TGFβ-induced fibroblast activation (27). Moreover, extracellular matrix deposition was reduced by rapamycin, which inhibits the expression of the plasminogen activator inhibitor-1 and TGFβ (28). Lastly, Xu et al. (29) demonstrated that inhibition of mTOR can suppress the epithelial-mesenchymal transition induced by TGF-β1.

Both inflammation process and neoangiogenesis can directly influence the development of fibrosis because of the release of profibrotic factors and the transition of recruited pericytes to a fibroblast phenotype. Both processes interact to modulate scar formation (30). According to clinical observations, the fibrotic regions show reduced vascularization, while increased vascular remodeling is observed in normal lung parenchyma. One possible explanation for these alterations in vascular architecture is an unbalanced production of proangiogenic and antiangiogenic mediators. Similar to those discovered in healing wounds, pro- and antiangiogenic mediators are present in the fibrotic lung and could lead to vascular injury, epithelial cell proliferation, and pulmonary fibrosis lesions (31).

Our findings are consistent with the observation that the presence of mTORi improves pulmonary perfusion and therefore may have a potential role in slowing or reducing the development of fibrotic pulmonary lesion. Potential study limitations include the relatively small number of patients, other than the fact that none of the fibrosis markers have been evaluated.

Then, we are aware that the observed improvement in pulmonary perfusion within the mTORi group can suggest, but not definitively confirm, a beneficial effect of mTORi, however, our conclusions are based on several factors. First, the study of the pulmonary perfusion was assessed at two distinct time points (1 and 6 months after recovery) and it has been designed to allow each patient to act as their own control in the attempt to minimize the inter-patient differences and confounding factors, including pre-existing alterations of pulmonary perfusion. Moreover, the pre- and post-COVID-19 infection immunosuppressive therapy was not modified in both groups. We think that comparing pulmonary perfusion within the same individuals in these comparable groups helps to assess the impact of different immunosuppressive regimens.

In conclusion, while we cannot exclude the possibility that the observed pulmonary perfusion changes could reflects the natural course in non-immunosuppressive patients, further studies are needed, we think that our findings should be interpreted within the specific context of the kidney transplanted population and the comparison between the two transplanted groups could provide valuable insights.

Moreover, we are aware that the study has statistical limitations. While propensity score matching (PSM) was used to reduce confounding factors, several limitations remain, particularly due to the small sample size. Residual confounding from unmeasured variables is still possible. Adequate overlap of propensity scores between groups was confirmed, and a sensitivity analysis was performed to assess the robustness of results, which remained consistent. Covariates were selected based on clinical relevance and literature, aiming to reduce confounding by indication. Nearest-neighbor 1:1 matching without replacement was used to avoid oversampling and ensure reliable inference. Despite these precautions, limitations inherent to PSM must be considered when interpreting the findings.

Finally, our observations represent, to the best of our knowledge, the first attempt to investigate the effects of the modulation of chronic immunosuppression during and after COVID-19 infection in terms of improvement in pulmonary perfusion mediated by chronic pharmacological mTOR inhibition.

## Data Availability

The raw data supporting the conclusions of this article will be made available by the authors, without undue reservation.
