# Supplementary material for: Impact of immunosuppressive therapy on pulmonary perfusion in kidney transplant recipients after COVID-19 illness
Source: Front Med (Lausanne). 2025 Jun 11;12:1562407. doi: 10.3389/fmed.2025.1562407 (PMC12187664; doi:10.3389/fmed.2025.1562407)
Supplement: Supplementary file 1 [file Table_1.docx]

**Supplementary Table 1**

|  | **Total** | **Group A**  **(Tac/MMF/Pred)** | **Group B**  **(Tac/mTORi/Pred)** | ***P*** |
| --- | --- | --- | --- | --- |
| Number (n) | 93 | 49 | 44 |  |
| Gender (% female) | 28 (30.1%) | 16 (32.6%) | 12 (27.2%) | 0.356 |
| Age (years) | 54.3±13.2 | 59.5±14.1 | 49.8±15.2 | 0.049 |
| Time from transplantation (months) | 106.0±42.1 | 128.2±39.1 | 84.0±72.5 | 0.036 |
| eGFR (ml/min) | 50.8±17.4 | 53.9±19.5 | 47.3±16.9 | 0.058 |
| Diabetes Mellitus (%) | 11 (11,8%) | 5 (10.2%) | 6 (13.6%) | 0.609 |

*Note*: Values are expressed as mean ± SD, counts (*n*), or percentages (%).

Abbreviations: eGFR. estimated glomerular filtration rate; MMF. mycophenolate mofetil; mTOR. mTOR inhibitors; Pred. prednisolon; Tac. tacrolimus.

**Supplementary Figure 1: Love plot showing the standardized mean difference (SMD) before and after PS‐matching**


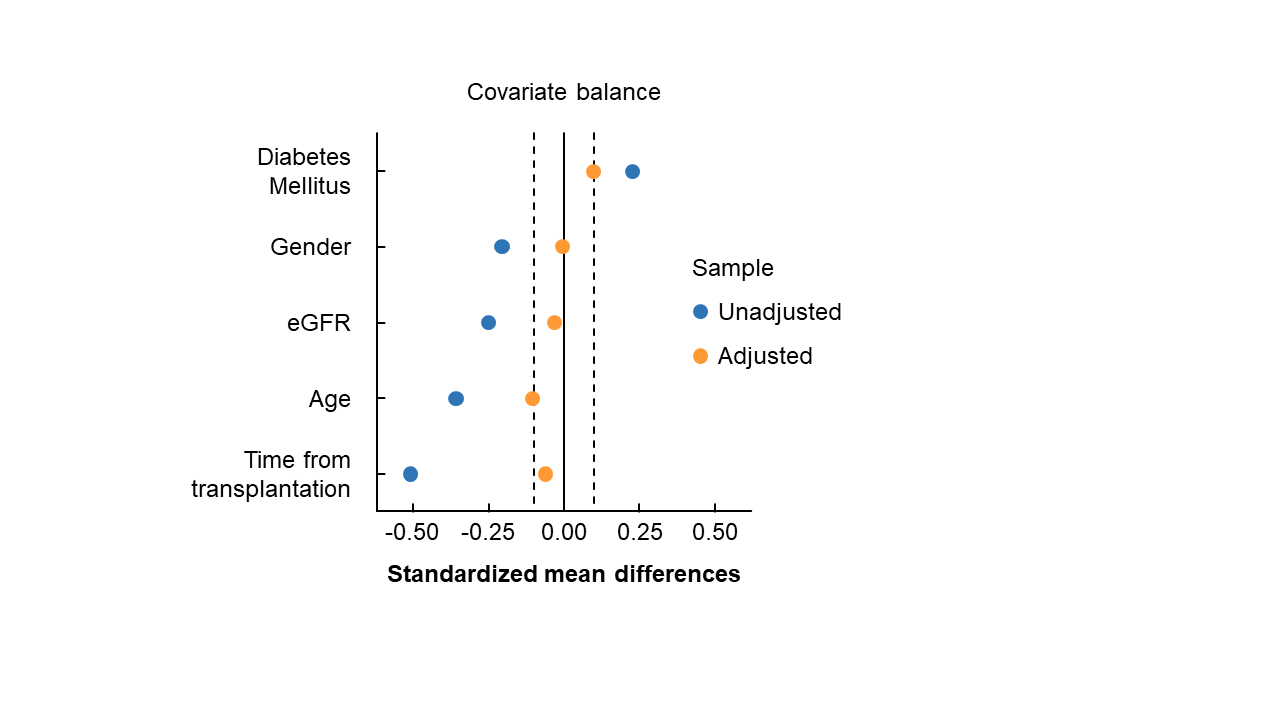


Love plot analysis displaying the standardized mean differences (SMDs) for each covariate (presence of Diabetes Mellitus diagnosis, Gender, estimated GFR, age at transplantation, time from transplantation) before and after propensity score matching. The vertical dotted line at 0.1 represents the commonly accepted threshold for adequate balance. Covariates with post-matching SMDs below this threshold are considered well-balanced between groups. All covariates achieving SMDs < 0.1 after matching indicate successful reduction of baseline differences and appropriate covariate balance.

Abbreviations: eGFR. estimated glomerular filtration rate
